# Effects of Shift Work on Cognitive Performance, Sleep Quality, and Sleepiness among Petrochemical Control Room Operators

**DOI:** 10.5334/jcr.134

**Published:** 2016-02-03

**Authors:** Reza Kazemi, Rashid Haidarimoghadam, Majid Motamedzadeh, Rostam Golmohamadi, Alireza Soltanian, Mohamad Reza Zoghipaydar

**Affiliations:** Phd student of occupational health, occupational health department, school of heath, Hamadan University of Medical Sciences, Hamadan, Iran; Ergonomics Department, Hamedan University of Medical Sciences, Hamadan, Iran; Department of Occupational Hygiene, University of Medical Sciences, Hamadan, Iran; Department of Biostatistics and Epidemiology, Hamadan University of Medical Sciences, Hamadan, Iran; Department of Psychology, Buali Sina University, Hamadan, Iran

**Keywords:** shift work, cognitive performance, sleepiness, sleep quality, control room

## Abstract

Shift work is associated with both sleepiness and reduced performance. The aim of this study was to examine cognitive performance, sleepiness, and sleep quality among petrochemical control room shift workers. Sixty shift workers participated in this study. Cognitive performance was evaluated using a number of objective tests, including continuous performance test, n-back test, and simple reaction time test; sleepiness was measured using the subjective Karolinska Sleepiness Scale (KSS); and sleep quality was assessed using the Pittsburgh Sleep Quality Index (PSQI) questionnaire. ANCOVA, t-test, and repeated-measures ANOVA were applied for statistical analyses, and the significance level was set at *p* < 0.05. All variables related to cognitive performance, except for omission error, significantly decreased at the end of both day and night shifts (*p* < 0.0001). There were also significant differences between the day and night shifts in terms of the variables of omission error (*p* < 0.027) and commission error (*p* < 0.036). A significant difference was also observed between daily and nightly trends of sleepiness (*p* < 0.0001) so that sleepiness was higher for the night shift. Participants had low sleep quality on both day and night shifts, and there were significant differences between the day and night shifts in terms of subjective sleep quality and quantity (*p* < 0.01). Long working hours per shift result in fatigue, irregularities in the circadian rhythm and the cycle of sleep, induced cognitive performance decline at the end of both day and night shifts, and increased sleepiness in night shift. It, thus, seems necessary to take ergonomic measures such as planning for more appropriate shift work and reducing working hours.

## 1. Introduction

Shift work is usually referred to as dividing working hours among two or more occupational groups in order to cover the time needed for duty performance or for production process. Shift workers are those who perform duties outside the regular working hours before 7 am and after 6 pm [1]. Evidence shows that some of the industry-related accidents during the last few decades, such as Chernobyl, Three Mile Island, Bhopal, and chemical spill into Rhine and Valdez, have all occurred in the middle of the night, and, according to the investigations, the main cause of these accidents was reported to be shift work and human errors committed by control room operators [2,3]. Cognitive functions, such as working memory, attention, information processing speed, etc., play a vital role in the performance of many tasks; therefore, even temporary failure of cognitive and mental performance can lead to serious consequences for people, especially when accurate and immediate response is required [4]. Results obtained from field and laboratory studies have shown that shift work can alter circadian rhythms, disrupt the sleep cycle, and hinder human performance [5]. Laboratory studies have also revealed a wide range of cognitive declines caused by lack of sleep. Sleep deprivation makes people feel sleepy and also reduces alertness level. It may also lead to disorders of cognitive performance such as reduced ability to concentrate, increased reaction time, decreased ability to learn and recall new facts, and impaired motor skills [6]. In addition, sleep deprivation can be responsible for irritability, loss of communication skills, and lack of adaptation to the emotional needs in the workplace. Scholars have also reported that sleep deprivation reduces the ability to make sound decisions, thus increasing risk behaviors [7]. Reduction or failure of neurocognitive functions also results in an increase in the number of injuries caused by fatigue and occupational errors [8]. Moreover, research suggests that sleep deprivation and disruption of circadian rhythms could lead to attention failures and longer reaction time, hence increasing error rates and hindering performance [5]. The patterns of fixed shift work feature a higher likelihood of errors and accidents. Compared with the day shift, the risk increases by 15% on the evening shift and by 28% on the night shift. In addition, 10-hour and 12-hour shifts have, respectively, 13% and 28% higher risk in comparison with 8-hour shifts. The risk in consecutive shifts increases by 17% on the third night and 36% on the fourth night of the shift [6]. Investigating the effects of shift work on cognitive performance is complex since this can be affected by sleep disorders and other consequences of irregularities in the circadian cycle [5]. Some studies, especially in the laboratory setting, have shown a relationship between cognitive performance disorders and sleep disorders. Furthermore, the adverse effect of sleep disorders on executive functions (i.e. short-term memory, working memory, and attentional processes) have also been studied [5,8]. According to these studies, night work leads to a two-hour reduction of sleep and, thus, a reduction of sleep quality [8]. Research on shift truck drivers, medical residents, and pilots has demonstrated an increase in the accidents and human errors caused by sleep deprivation due to shift work [9]. Some studies have reported night shift residents’ and nurses’ concentration decline, which in turn reduces efficiency and enhances the number medical errors [10].

To the best of our knowledge, however, no study has focused on the effect of shift work on cognitive functions, sleepiness, and sleep quality of industrial workers, in general, and petrochemical control room operators, in particular. The current study aimed at addressing this gap in the literature by examining whether control room operators would experience detectable declines in cognitive performance, sleepiness, and sleep quality following the day and night shifts. Of particular interest to the current study was investigating the control room operators’ sustained attention, working memory, reaction time, sleepiness, and sleep quality during the day and night shifts.

The following hypotheses were formulated in the present study:

1. The cognitive functions would significantly decrease at the end of the day and night shifts, and the cognitive functions would be better during the day rather than the night shift.
2. Sleepiness trend would be higher for the night shift rather than the day shift, and people would suffer more from sleepiness during the night shift.
3. The quality of night sleep would be better than day sleep when people are on the day shift.

## 2. Materials and Method

### 2.1 Participants and design

The participants were 60 male control room operators working in the Petrochemical Complex, the largest petrochemical center located in southern Iran. The exclusion criteria included current use of hypnotic drugs, psychiatric illness, major systemic disease, and sleep disorders. Caffeine use was not restricted for the purpose of the study.

### 2.2 Study protocol

The participants’ work schedule included seven consecutive night shifts, seven consecutive day shifts, and seven consecutive days off. The day shift started at 0700 h and ended at 1900 h, while the night shift started at 1900 h and ended at 0700 h. The participants first completed a background questionnaire asking for demographic information. For assessing participants’ cognitive performance and subjective sleepiness, they were tested 30 minutes before and immediately following the night shift. The participants also completed the Karolinska Sleepiness Scale (KSS) every two hours during the day and night shifts. In addition, the Pittsburgh Sleep Quality Index (PSQI) questionnaire was used to measure workers’ sleep patterns (i.e., sleep quantity and quality during the opposite shift work). During the cognitive performance testing, participants were seated in a quiet room away from their individual workplace, with an experimenter being present in order to supervise the tests. To observe ethical concerns, a written consent form was obtained from all the participants prior to the study.

### 2.3 Cognitive tests

#### 2.3.1 N-back test (working memory test)

N-Back test, a frequently used measure of working memory, was employed in the current study. This instrument assesses the ability to process, select, and store information within a very short period of time. A total of 120 digits were displayed one by one on the center of the screen for five minutes, with an interval of 1500 milliseconds (ms). Participants were asked to compare the two consecutive digits that appeared on the screen and press a response button on a special keyboard as soon as the two digits were the same. The number of correct and false responses and the reaction time (in ms) were recorded as the dependent variables.

#### 2.3.2 Continuous performance test (sustained attention test)

Continuous performance test was used as a measure of attention error and sustained attention. A validated Persian version of the instrument was employed in the present study. The test consists of 150 stimuli appearing on the screen; the participants’ task was to press the spacebar on their keyboards as soon as the number ‘‘4’’ was displayed on the screen. The presentation time for each stimulus was 150 ms, with an interval of 500 ms between every two stimuli. The number of correct responses, omission error, commission error, and the reaction time (in ms) were recorded as the dependent variables.

#### 2.3.3 Reaction time test

Reaction time relates to the speed at which the information is processed. The reaction time test which was employed in the current study has been validated as a measure of cognitive performance [11]. The test includes black squares that appear on the screen at random intervals (i.e., the stimulus). Participants were instructed to respond to this stimulus as soon as possible by pressing a key. The reaction time was recorded in milliseconds. If no response was made within 1750 ms, a new interval would start. If the participant pressed the key before the start of the stimulus or within 120 ms following it, the response was ignored, and a warning signal was shown. Mean and standard deviation of reaction times (ms) were used in the analysis as the statistical parameters.

#### 2.3.4 Sleepiness (Karolinska Sleepiness Scale)

The Karolinska Sleepiness Scale (KSS) was used in the present study as a self-declaration measure of sleepiness. The instrument, which enjoys a fairly good level of reliability and validity [12], is based on a nine-point scale including 1 (*very alert*), 3 (*alert*), 5 (*neither alert and nor sleepy*), 7 (*sleepy*), and 9 (*very sleepy and trying to stay awake*).

#### 2.3.5 Sleep quality (Pittsburgh Sleep Quality Index questionnaire)

Sleep duration and sleep quality were measured for day shifts off and night shift off by the Pittsburgh Sleep Quality Index (PSQI) questionnaire. The instrument items include ‘*When you are working day shifts and night shifts, how many hours do you usually sleep during the off-duty period?*’ and ‘*How well do you assess your sleep quality during this period?*’

#### 2.3.6 Statistical Analysis

Within and between group sleep trend differences were assessed through repeated measurement Analysis of Variance (ANOVA). Mauchly’s test was also used as the test of Sphericity. Furthermore, t-test was applied to compare the value of sleepiness in each measurement between the two groups. Also, the mean score of sleep quality and its components were compared between the two groups through t-test. Analysis of Covariance (ANCOVA) was used to evaluate the changes in the participants’ cognitive performance before and after the shift. Variable values measured before the shift entered the ANCOVA model as covariates and the ones obtained after the shift were compared between the two work shifts. All statistical analyses were conducted through SPSS (IBM Corporation, Armonk, New York), with the significant P-value considered to be 0.05.

## 3. Results

Participants’ mean age and working experience were 30.1 ± 2.46 and 6.4 ± 1.36 years, respectively. In terms of academic degree, the majority of participants (48.3%) were Bachelor degree holders, 15% held Masters, and the rest had Associated Diploma.

**Figure 1:**
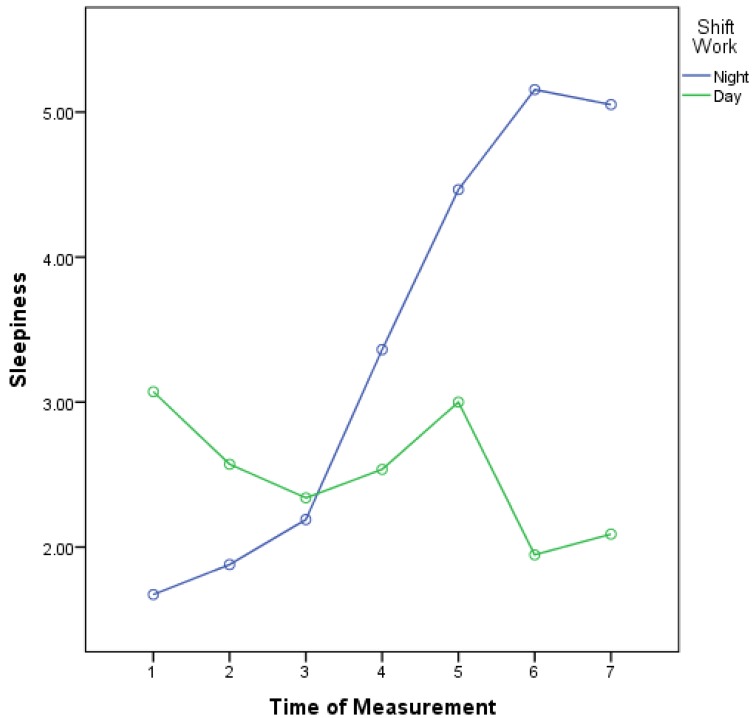
Trend of sleepiness in day-shift and night-shift workers over seven consecutive segments of the work shift.

The results of paired sample t-test (Table 1) showed that, except for the omission error among night shift participants, a significant difference was observed in all the other variables between before and after night shift measurements. In addition, except in the case of omission error and CPT response time, the changes of all variables were statistically different before and after day shift. Moreover, the results obtained from ANCOVA (Table 2) indicated that there was a significant difference between the two shifts in change of omission and commission errors before and after each shift (P < 0.05). Nevertheless, the changes in N-back score, N- back response time, CPT response time, and Reaction time were not statistically different between the two shifts (P > 0.0.5). As illustrated in Table 3, there was a significant trend in sleepiness among different measures in the two shifts (P < 0.001). The sleepiness trend in the night shift was increasing, while it was decreasing in the day shift. Moreover, save for the third measurement (23:00/11:00), all measures were different between subjects of the two shifts (P < 0.001). In addition to the overall trend of sleepiness, the Greenhouse-Geisser test showed a significant difference between the trend of two shifts. Figure 1 demonstrates the line trend based on the two shifts. Figure 1 shows an inverse trend of sleepiness in the two shifts, with a crosspoint in the third measurement.

**Table 1:** Comparing of mean and standard deviation of cognitive variables before and after shift in two work shifts.

Variables		Night shift		P value*	Day shift		P value*
		Before	After		Before	After	

N-back	Score	104.8 ± 8.1	94.6 ± 6.3	<0.001	106.1 ± 7.9	102 ± 6.74	<0.001
	Response time	663 ± 102	730.5 ± 79.4	<0.001	687.3 ± 104.5	729.5 ± 87	<0.001
CPT	Omission error	0.350 ± 0.61	0.433 ± 0.72	0.520	0.183 ± 0.50	0.217 ± 0.524	0.709
	Commission error	0.68 ± 0.66	1.55 ± 1.13	<0.001	0.4 ± 0.56	0.95 ± 0.83	<0.001
	Response time	419.9 ± 25.6	438.5 ± 31.8	<0.001	411.6 ± 19.7	418 ± 21.9	.141
Reaction time		227.5 ± 28.4	244/4 ± 34.7	<0.001	227.6 ± 28.3	254.8 ± 38.5	<0.001

**Table 2:** Comparing of mean and standard deviation of cognitive variables between night and day shift work.

Variables		Night shift	Day shift	P value*

N-back	Score	94.6 ± 6.27	102 ± 6.74	0.388
	Response time	730.5 ± 79.4	729.5 ± 86.9	0.966
CPT	Omission error	0.433 ± 0.72	0.217 ± 0.52	0.027
	Commission error	1.55 ± 1.12	0.95 ± 0.83	0.036
	Response time	438.5 ± 31.8	417.9 ± 21.9	0.058
Reaction time		244.4 ± 34.7	254.8 ± 38.5	0.398

*ANCOVA test.

**Table 3:** Mean and standard deviation of sleepiness in seven repeated measures in two work shifts.

	Night shift	Day shift	P value^‡^

Sleepiness 1	1.67 ± 0.89	3.07 ± 1.78	<0.001
Sleepiness 2	1.88 ± 0.86	2.57 ± 1.45	0.001
Sleepiness 3	2.19 ± 1.28	2.34 ± 1.19	0.521
Sleepiness 4	3.36 ± 1.38	2.54 ± 1.59	0.001
Sleepiness 5	4.46 ± 1.82	3 ± 1.41	<0.001
Sleepiness 6	5.15 ± 2.08	1.95 ± 0.92	<0.001
Sleepiness 7	5.05 ± 2.27	2.09 ± 1.16	<0.001
P value^†^	<0.001	<0.001	

^‡^ t-test. ^†^ repeated measure for ANOVA.

Sleep quality and quantity, as well as different components of sleep quality, were compared between the two shifts by t-test. The results (Table 4) showed that sleep quality and sleep quantity values were statistically different between the workers of the two shifts (P < 0.01). More specifically, the sleep quality scores were significantly higher among night-shift-off workers than their day-shift-off counterparts. In contrast, day-shift-off participants scored significantly higher in the sleep quantity index compared to their night-shift-off colleagues. No significant difference was observed between the two groups with respect to sleep quality components, including sleep latency, sleep duration, sleep efficiency, sleep disturbances, sleeping medication, and dysfunction (P > 0.05).

**Table 4:** Mean and standard deviation of sleep quality and different components in two work shifts.

	Day off	Night off	P value^‡^

Sleep quality	1.63 ± 0.80	2.22 ± 0.67	0.001
Sleep latency	1.83 ± 0.81	1.75 ± 0.86	0.584
Sleep duration	1.52 ± 0.79	1.57 ± 0.93	0.751
Sleep efficiency	0.62 ± 0.84	0.87 ± 0.96	0.134
Sleep disturbances	1.25 ± 0.79	1.25 ± 0.89	0.999
Sleeping medication	0.13 ± 0.34	0.18 ± 0.43	0.484
dysfunction	1.38 ± 0.74	1.37 ± 0.71	0.90
PSQI score	8.32 ± 3.41	9.03 ± 3.1	0.230
Sleep quantity	6.97 ± 1.08	5.83 ± 0.92	<0.001

^‡^ t-test.

## 4. Discussion

### 4.1 Cognitive performance

One of the aims of the present study was to compare cognitive functions at the beginning and end of the day and night shifts. Any change in at least one of the variables in each test, when compared with the respective values in the beginning of the shift, could indicate a failure of cognitive performance. According to the results, the reaction time and the number of commission errors in the CPT test increased at the end of both shifts, the number of correct responses in the n-back test decreased, and the reaction time increased at the end of both shifts. In other words, all three cognitive functions under study (viz., working memory, sustained attention, and reaction time) were impaired and showed a significant decrease at the end of both day and night shifts. Additionally, the comparison of these functions on day and night shifts revealed that omission errors, commission errors, and the speed of response in the sustained attention test were significantly higher on the night rather than the day shift, indicating the greater vulnerability of attention on the night shift. The obtained results were consistent with the first hypothesis that predicted a decline in cognitive performance at the end of both shifts and a lower level of performance on the night shift in comparison with the day shift. Although these variables have not been examined in a field study in previous research, the findings of the current study were generally in line with those reported in previous studies regarding cognitive performance [5,13,14,15]. In Machi et al.’s study conducted among emergency physicians, short-term memory showed a significant decrease at the end of both day and night shifts [16]. This decline could be attributed to irregularities of circadian rhythmicity in shift workers as well as the fatigue caused by long working time [16]. As demonstrated in several studies, fatigue is among the main disadvantages of 12-hour shifts that can impair cognitive performance, reduce alertness level, and increase the risk of accidents [17]. The results of this study also showed that the parameters related to attention would be more negatively affected on the night rather than the day shift. The reason may lie in the relationship between cognitive performance and frontal lobe as well as the frontal part of the brain that is vulnerable to sleep deprivation, in general, and night sleep, in particular [18].

### 4.2 Sleepiness

The second aim of this study was to compare the sleepiness trend on the day and night shifts. The results indicated a steadily increasing sleepiness trend for the night shift and a stable or decreasing one for the day shift. In general, the trend for the second half of the night shift reached its peak and was significantly higher than that for the day shift except for the beginning of the shift in which the sleepiness trend for the day shift was higher than that for the beginning of the night shift. Although we were unable to locate a study similar to the present one, previous research has also reported an above-average sleepiness trend for shift workers, in general, and night workers, in particular [19,20]. In addition, many studies have shown that shift workers suffer from extreme sleepiness, and about 32% of them also suffer from insomnia as well as extreme sleepiness during the day, a result that is close to the findings of the present study. Increased sleepiness during the night shift is related to the natural circadian rhythmicity of sleepiness and indicates lack of adaptation of the body to night work. This can threaten the safety of work due to the sensitive nature of such occupations because people require adequate alertness during their shift time in order to manage and control inherently dangerous chemical processes [21]. Therefore, measures need to be taken in this regard in order to reduce sleepiness and increase alertness on the night shift. The higher sleepiness rate at the beginning of the day shift can be attributed to the early start of the shift time because people need to wake up and attend work at 6 o’clock in the morning. Given the 12-hour shift, people have little time to sleep at night, making them suffer from sleepiness at the early hours of the shift. In the course of time, their sleepiness level would decrease to the normal level. In the present study, a comparison was made between day and night shift workers’ sleepiness level, and no significant difference between the day and night sleepiness was expected considering the participants’ appropriate history of shift work (they had at least 12 times of shift work within a month) and the adaptation of their circadian rhythm; however, the results were inconsistent with the formulated hypothesis. Previous studies have shown that for those who were awake for several consecutive nights, the sleepiness level decreased and, then, got back to the normal level, a result that is in contradiction to the findings of the current study [22]. The reason may be the fast rotation from the day shift to the night shift and, hence, lack of adaptation to night work.

### 4.3 Sleep quality

We found that the mean final scores of PSQI related to the day and night sleep were 8.32 and 9.03, respectively, which are higher than the cut-off point reported by other scholars. This indicates low sleep quality in the studied sample. Buysse et al. reported less than five and Backhaus et al. considered less than six as the cut-off points for desirable sleep quality [23]. No significant difference between the day and night shifts were observed with respect to the final PSQI scores; however, subjective sleep quality and quantity on the day and night shifts were significantly different in the sense that sleep quality and quantity were lower during the day rather than the night shift. The results were in line with those reported in other studies. Machi et al used PSQI questionnaire to evaluate sleep quality among shift emergency physicians and indicated that a significant percentage of the participants suffered from poor sleep quality [15]. Shortening sleep time creates involuntary episodes, called Micro Sleep, that last 10 to 15 seconds. During these episodes, memory and alertness are impaired, resulting in errors while working [24]. In addition, research into shift workers has shown that such workers sleep from 15% to 20% less than other workers, and the duration of their sleep was about 1.5 to 9.5 hours a day [25]. This is in line with the results observed in the current study. The reduction of sleep duration in shift workers, irregularities in the circadian rhythm, and the great length of working time per shift (so that people spend around 13 to 14 hours for each shift, including transportation time) could be regarded as factors responsible for the poor quality and inadequate quantity of sleep among the participants in the present study.

## 5. Conclusions

Overall, the study showed that, due to long working hours of shift workers and the resultant fatigue, there was a significant reduction in cognitive functions at the end of both day and night shifts. On the other hand, cognitive performance was impaired more during the night shift. These findings are related to the irregularities in the circadian rhythm and the natural cycle of sleep and waking, and also lack of adaptation of circadian rhythm to the new conditions. Moreover, the rate of sleepiness during the night shift was higher than that for the day shift, and the sleep quality among the participants was low, which was dependent on long working time and short resting time. The tasks assigned to the studied sample require qualities such as adequate alertness and consistent performance. If the people do not possess these qualities, the safety of work would be at risk [21]. In view of these findings, we suggest that preventive measures (e.g. reducing working hours, improving lighting in the work environment, and letting staff members take a nap during the shift) should be taken. Of course, reducing working hours can impose undue costs on the organization and, therefore, may not be easily accepted by managers. Improving the lighting condition and taking naps especially during night shifts, on the contrary, can be useful ergonomic strategies. Previous studies have indicated that bright light improves individuals’ cognitive performance, especially working memory, sustained attention, and alertness. Furthermore, studies have shown that, as an appropriate strategy which is becoming popular among shift workers, taking naps during the shift improves alertness and removes fatigue [17,26].

## Competing Interests

The authors declare that they have no competing interests.
